# Time to Positivity in Blood Culture Bottles Inoculated with Sonication Fluid from Fracture-Related Infections

**DOI:** 10.3390/microorganisms12050862

**Published:** 2024-04-26

**Authors:** Leopold Henssler, Lena Schellenberger, Susanne Baertl, Lisa Klute, Robert Heyd, Maximilian Kerschbaum, Volker Alt, Daniel Popp

**Affiliations:** 1 Department of Trauma Surgery, University Hospital Regensburg, 93053 Regensburg, Germany; 2 Institute of Clinical Microbiology and Hygiene, University Hospital Regensburg, 93053 Regensburg, Germany

**Keywords:** sonication, fracture-related infections, time to positivity, microbiology, diagnostics, antibiotic stewardship, bacteria

## Abstract

The timely and accurate identification of causative agents is crucial for effectively managing fracture-related infections (FRIs). Among various diagnostic methods, the “time to positivity” (TTP) of cultures has emerged as a valuable predictive factor in infectious diseases. While sonication of implants and inoculation of blood culture bottles with sonication fluid have enhanced sensitivity, data on the TTP of this microbiological technique remain limited. Therefore, patients with ICM criteria for confirmed FRI treated at our institution between March 2019 and March 2023 were retrospectively identified and their microbiological records were analyzed. The primary outcome parameter was TTP for different microorganism species cultured in a liquid culture collected from patients with confirmed FRI. A total of 155 sonication fluid samples from 126 patients (average age 57.0 ± 17.4 years, 68.3% males) was analyzed. Positive bacterial detection was observed in 78.7% (122/155) of the liquid culture pairs infused with sonication fluid. *Staphylococcus aureus* was the most prevalent organism (42.6%). Streptococcus species exhibited the fastest TTP (median 11.9 h), followed by *Staphylococcus aureus* (median 12.1 h) and Gram-negative bacteria (median 12.5 h), all of which had a 100% detection rate within 48 h after inoculation. Since all Gram-negative pathogens yielded positive culture results within 24 h, it could be discussed if empirical antibiotic therapy could be de-escalated early and limited towards the Gram-positive germ spectrum if no Gram-negative pathogens are detected up to this time point in the context of antibiotic stewardship.

## 1. Introduction

Fracture-related infection (FRI) is one of the most devastating and challenging complications following fracture treatment. For patients, FRI often leads to permanent loss of function [1] or even amputation. At the same time, the socioeconomic burden caused by these cost-intensive treatments [1,2] has emerged with increasing incidence over the last decade [3]. Thus, the prompt and precise identification of infection and determination of causative agents is imperative for managing patients with a suspected FRI. Nonetheless, the diverse clinical presentations of FRI pose challenges in distinguishing them from non-infectious etiologies. To enhance diagnostic accuracy, an international panel of experts established diagnostic criteria for an FRI in 2018 [4]. According to this consensus, confirmation of an FRI diagnosis via tissue biopsies during surgical revision is necessary in suspected cases lacking indicative clinical signs like fistulae, sinus tracts, or purulent wound drainage [4,5]. Biofilm formation, invasion of the canaliculi within the bone matrix [6], formation of staphylococcal abscess communities [7], and intracellular infections [8] further complicate both the diagnosis and treatment of these infections. Utilizing sonication of removed orthopedic implants has significantly improved the diagnostic sensitivity compared to conventional tissue biopsies [9,10]. Sonication involves employing ultrasound energy to dissolve bacteria from the biofilm on the implant, followed by a culture-based analysis of the sonicate fluid [11]. Additionally, inoculating blood culture bottles with sonication fluid has proven to further enhance the sensitivity compared to a conventional culture of the sonicate fluid [12].

The time to positivity (TTP) of cultures refers to the duration between specimen incubation and the first signal of microbial growth. Apart from microbiological diagnostic sensitivity, a short time to positivity of microbiological culture methods is crucial for the precise treatment and early administration of appropriate antibiotics. However, the TTP in tissue cultures from periprosthetic joint infections (PJI) displays high variability between causative pathogens [13] and acute or chronic infections [14]. In particular, slowly growing microorganisms typically exhibit a prolonged TTP in conventional cultures [13]. Hence, the international consensus meeting on FRI recommends an extended incubation of tissue samples from FRI and PJI for 10 to 14 days [5]. However, this prolonged incubation period poses the risk of delayed diagnosis, inappropriate empirical antibiotic therapy, and elevated rates of adverse drug effects [15] during the immediate postoperative phase. Moreover, the literature has demonstrated a predictive capability of the parameter of TTP for clinical outcomes and its utility in guiding antimicrobial therapy in bacteremia cases [16,17,18].

Since data are scarce on the TTP of causative pathogens in cultures obtained from FRI cases, this study aimed to investigate the TTP of microorganisms isolated by inoculating sonication fluid into blood culture bottles in patients undergoing revision procedures with orthopedic implant removal for treatment of FRI. In particular, the influence of different microbial species, different blood culture bottles, implants, and patient-related factors on the TTP were tested in this study to investigate if TTP can be regarded as a characteristic property of microorganisms and could therefore influence treatment decisions.

## 2. Materials and Methods

### 2.1. Study Population

Patients treated for fracture-related infections (FRIs) at our institution between March 2019 and March 2023 were identified retrospectively using the hospital’s patient data management system. For this, research personnel scanned all surgical reports and outpatient documentation for eligible patients. For confirmation of the FRI diagnosis, the 2018 ICM criteria for FRI [4] were applied. This assessment was performed using data collected on serum and synovial fluid biomarkers, culture results, histology results, clinical reports, and intraoperative findings. Following this procedure, we were able to identify 162 patients with ICM criteria-confirmed FRI. Of the total 162 patients with diagnosed FRI, 155 sonication fluids of 126 patients were identified for further analysis. In addition to microbiological records, each patient’s medical records were reviewed, and patient demographic characteristics (age, sex) and comorbidities (ASA score, diabetes, arterial occlusive disease) were extracted. In addition, surgical data on the affected implant, bone or joint and laterality were obtained. The study protocol was approved by the Institutional Ethics Committee of the University (ref. number 23-3359-104, 2 May 2023) prior to conduction of the analysis.

### 2.2. Sample Collection and Microbiological Methods

All patients had undergone implant removal or exchange during the surgical treatment. For sonication-based microbiological testing, all orthopedic implants were transferred to the local Institute of Clinical Microbiology and Hygiene in sterile single-use boxes. Under a laminar flow hood, the material was overlayed with phosphate-buffered saline (PBS) and sonicated within an ultrasound bath (BactoSonic, Bandelin, Berlin, Germany) at 40 kHz (200 W) for 5 min. Thereafter, the sonication fluid was centrifuged (4600× *g*, 15 min) and the supernatant was discharged. The remaining bottom layer (ca. 3 mL) was resuspended and inoculated in blood culture bottles (pediatric and lytic anaerobic, 1 mL each; BACTEC FX, BD, Heidelberg, Germany) and standard growth media (fluid: thioglycolate and brain heart infusion broth; solid: chocolate blood, fresh blood, and Schaedler agar). Cultures were incubated under standard aerobic and anaerobic conditions and evaluated for growth up to 14 days after inoculation. Bacterial identification was performed by MALDI-TOF mass spectrometry (Bruker Daltonics, Bremen, Germany) and antibiotic susceptibility testing was conducted using a Phoenix M50 system (BD, Heidelberg, Germany) with results being reported according to EUCAST rules.

### 2.3. Outcome Parameters

The primary outcome parameter of this analysis was the TTP for different microorganism species cultured in liquid culture of sonication fluids that were collected from patients with confirmed FRI as diagnosed using the ICM 2018 criteria. We also investigated if the TTP varied in sonication fluids from different implants and in patients with or without common comorbidities.

### 2.4. Statistical Analysis

Metric data were analyzed and presented either as the mean and the standard deviation (M ± SD) or as the median and the interquartile range (IQR). Categorical data were presented as the total count and the percentage. Shapiro–Wilk test did not show normality of data regarding the time to positivity. Therefore, times to positivity of the same microorganism in different bottles were compared using the Wilcoxon test. Comparisons between independent groups were conducted using the Mann–Whitney U test. The statistical analysis was carried out using the SPSS software package Version 29.0 (SPSS Inc., Chicago, IL, USA). The level of significance was set at *p* < 0.05.

## 3. Results

### 3.1. Demographic Data

A total of 155 sonication fluids of 126 patients (see Table 1) was included in the analysis. Sonication was performed from screws in 73.5% of cases (114/155), plates in 34.2% (53/155), intramedullary nails in 12.9% (20/155), polymethylmethacrylate (PMMA) spacers in 4.5% (7/155), and spinal rods in 3.9% (6/155).

### 3.2. Microbiological Findings

Positive bacterial detection was observed in 78.7% (122/155) of the liquid culture pairs infused with sonication fluid. Comparing the rate of positive cultures in sonicated im-plants, positive cultures were obtained with this method in all spinal rods, 83.0% of plates (44/53), 82.5% of screws (94/114), and 75.0% (15/20) of intramedullary nails, while the lowest culture positivity rate was recorded in the sonication of PMMA spacers (4/7).

#### 3.2.1. Pathogen Spectrum

A total of 173 germs was detected in these 122 cases with positive cultures, leading to polymicrobial infections in 33.6% of cases (41/122). Of all the detected microorganisms, 136 bacteria (78.6%) could be detected in both the PED and anaerobic blood culture bottle, 19 germs (11.0%) were only evidenced in the anaerobic, and 18 germs (10.4%) were only detected in the PED culture.

The most prevalent organism (see Table 2) in our patient population was *Staphylococcus aureus* (*n* = 52), followed by Gram-negative rods (*n* = 29), *Staphylococcus epidermidis* (*n* = 27), *Enterococcus species* (*n* = 26), other coagulase-negative *Staphylococcus* species (*n* = 17), Streptococcus species (*n* = 13), *Cutibacterium acnes* (*n* = 2), *Candida* species (*n* = 1), and others (*n* = 6). In accordance with their metabolism, Cutibacteria were the only bacteria that were exclusively evidenced in anaerobic cultures.

#### 3.2.2. Time to Positivity

Of all 122 samples with positive cultures, the median TTP was 12.6 (IQR 9.1–18.5) hours in PED bottles and 12.7 (IQR 10.2–19.2) hours in anaerobic bottles and did not significantly differ between the bottles (*p* = 0.339). Positivity of at least one of the culture pairs was observed within 24 h after inoculation in 107 cases (87.7%). Moreover, the median TTP did not differ in patients with or without type II diabetes (16.2 [IQR 10.7–20.1] hours vs. 12.7 [IQR 10.4–19.2] hours; *p* = 0.453) or arterial occlusive disease (13.7 [IQR 9.7–27.8] hours vs. 12.7 [IQR 10.3–19.1] hours; *p* = 0.551). The median TTP was significantly longer when PMMA spacers were sonicated compared to all other metal implants (*p* = 0.013; see Table 3).

**Figure 1:**
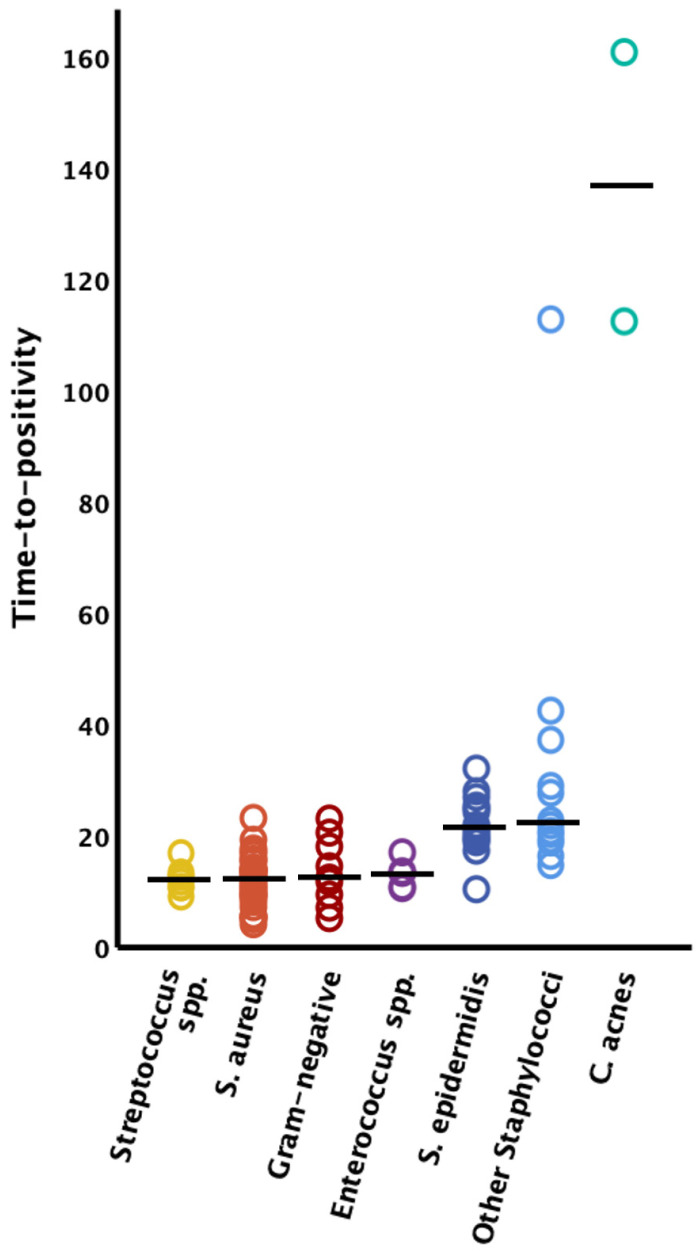
Time to positivity by microorganism type. The solid horizontal lines represent the median values; *S.* = *Staphylococcus*; spp. = species.

The time to positivity could only be clearly assigned to the causative germ in 97 bacteria in 95 cases; in the other cases there were polymicrobial infections where clear assignment of the TTP to the causative germ was not possible. Therefore, these latter cases had to be excluded from further analysis of the TTP. Streptococcus species demonstrated the fastest TTP (*n* = 8, median 11.9 h [IQR 10.5–12.9]), followed by *Staphylococcus aureus* (*n* = 37, median 12.1 h [IQR 9.0–13.9]), Gram-negative bacteria (*n* = 9, median 12.5 h [IQR 9.4–18.1]), *Enterococcus* species (*n* = 6, median 13.1 h [IQR 10.6–13.3]), *Staphylococcus epidermidis* (*n* = 18, median 21.3 h [IQR 19.2–24.7]), and other coagulase-negative Staphylococcus species (*n* = 14, median 22.2 h [IQR 19.2–37.1]). The longest latency to positive liquid cultures was observed in FRI caused by *Cutibacterium acnes* (*n* = 2, 112.4 h and 160.9 h) (see Figure 1).

**Figure 2:**
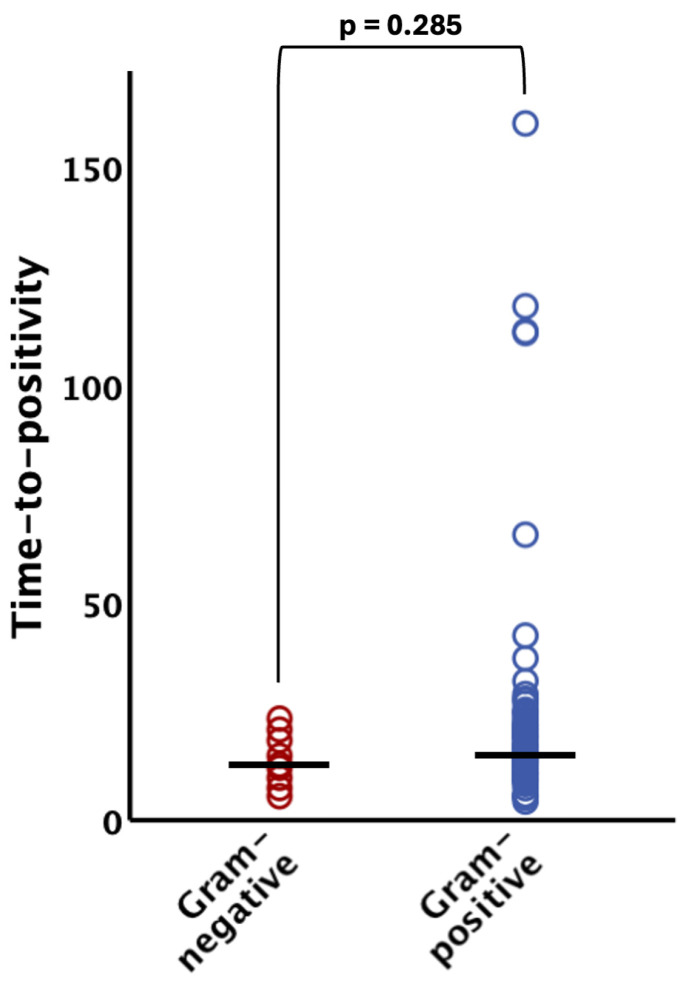
Time to positivity of Gram-negative and Gram-positive bacteria. There was no statistically significant difference (*p* > 0.05) between the groups. The horizontal lines indicate median values. The bracket indicates group comparison of time to positivity by Mann–Whitney U test.

The TTP did not significantly differ (*p* = 0.285) between Gram-negative organisms (median 12.5 h [IQR 9.4–18.1]) and their Gram-positive counterparts (median 14.9 h [IQR 10.6–21.3]) (see Figure 2). Notably, all Gram-negative bacteria were evidenced within 24 h after incubation of the blood culture bottles (95% CI 8.9–18.2 h; see Figure 1 and Figure 2).

## 4. Discussion

In this study, we investigated the microbiological findings and time to positivity in fracture-related infections using sonication fluid-infused blood culture bottles. Our results revealed the following key findings:

- Positive bacterial detection was achieved in 78.7% by incubation of sonication fluids into blood culture bottles in FRI cases and bacteria could be evidenced within 24 h in 87.7% of culture-positive cases.
- Streptococcus species, *Staphylococcus aureus*, and Gram-negative bacteria exhibited the shortest time to positivity (TTP) in our cohort, with 100% of these microorganisms being detected within 48 h after inoculation.
- Only *Cutibacterium acnes* and certain coagulase-negative Staphylococci were identified after more than 72 h

To our knowledge, this is the first study to report the TTP of common microorganisms evidenced by inoculation of sonication fluid into blood culture bottles in the treatment of fracture-related infections.

The technique of sonication of orthopedic implants for enhanced microbiological diagnostics in musculoskeletal infections was first described by Trampuz et al. [11] in 2007 and has proven valuable in the diagnostics of PJI in the past [19]. In the field of FRI, there is limited evidence that sonication fluid culture may be a useful adjunct to conventional tissue culture, but no strong evidence that it is superior or can replace a tissue culture [20]. Another promising analytic technique to increase the sensitivity in musculoskeletal infections is culturing tissue samples in blood culture bottles instead of a conventional culture [21]. Therefore, in this study, we tried to combine the potentially beneficial techniques of sonication and incubation in blood culture bottles and apply it to the challenging field of FRI to investigate the time to positivity of common microorganisms in these liquid cultures.

First, the sensitivity of this procedure was 79% in our study, which fits seamlessly into the reported range of sensitivity (65.9–94.7%) of conventional sonication fluid cultures but exceeds the sensitivity of conventional tissue cultures [20]. In order to increase the sensitivity of cultures, the present guidelines for the management of musculoskeletal infections recommend the incubation of bacterial cultures for at least 10–14 days [5,22]. Analyzing the TTP in microorganisms found in samples obtained from PJI, Tarabichi et al. [13] underlined the need for the prolonged incubation of conventional cultures, observing germ detection after more than 10 days, especially in low virulent and slowly growing pathogens [13]. An explanation for the long time to positivity in conventional cultures of periprosthetic or peri-implant tissue is that usually bacteria in these tissues are sessile in mature biofilms. Since bacteria in biofilms often have low replication rates compared to planktonic bacteria in tissue, the detection of bacteria in cultures from biofilms is usually more time-consuming. Therefore, various approaches have been pursued to accelerate microbiological diagnostics in musculoskeletal infections like PJI and FRI, in order to improve antibiotic stewardship by rapid de-escalation to targeted antimicrobial therapy, while at the same time providing a similar sensitivity as with a prolonged conventional tissue culture. Podleska et al. were able to demonstrate improved sensitivity by using the BACTEC™ blood culture system for the cultivation of intraoperatively harvested tissue samples [23]. Moreover, Peel et al. [21] could not only show improved sensitivity by incubating tissue samples in blood culture bottles, but also observed that the time to microorganism detection was shorter than with standard media (*p* < 0.0001). In this trial, aerobic and anaerobic blood cultures yielded positive results within a median of 21 and 23 h, respectively [21]. Conformingly, Minassian et al. [24] could reduce the duration of incubation required for the diagnosis of PJI using the BACTEC™ blood culture system for the incubation of tissue samples and found that incubation for more than three days failed to increase the sensitivity. Only *Cutibacterium* spp., coagulase-negative *Staphylococci*, and *Bacillus* spp. were detected thereafter [24]. These findings fit our present results in the related field of FRI. In our study, *Cutibacterium acnes* (112.4 h and 160.9 h), one *Staphylococcus hominis* (112.8 h), and one *Staphylococcus pettenkoferi* (228.5 h) were the germs with the longest TTP in our cohort and the only ones that were not detected within 72 h. We could show that the inoculation of blood culture bottles with sonication fluid from removed implants in FRI leads to positive germ detection after a median of 12.6 (IQR 9.1–18.5) hours in PED bottles and 12.7 (IQR 10.2–19.2) hours in anerobic bottles. In our cohort, positivity of at least one of the liquid cultures was observed within 24 h in 88% of cases, which is notably faster when compared to the conventional culture of soft tissue or bone samples [13], or even blood cultures from tissue samples [21] in the above-mentioned studies. Significantly, in our study, the median time to positivity was significantly prolonged in blood culture bottles inoculated with sonication fluid from PMMA spacers. This phenomenon may be caused by an increased elution of antibiotics from PMMA spacers following sonication [25], potentially inhibiting bacterial growth in the sonication fluid and thus prolonging the time to positivity of cultures.

The median TTP in our study did not differ between Gram-positive and Gram-negative bacteria. Although no Gram-negative pathogens were detected later than 24 h (95% CI 8.9–18.2 h) after incubation, some low-virulent Gram-positive bacteria were still detected after this time. This early detection of Gram-negatives prompts a discussion regarding an early modification of empirical antibiotic therapy in the context of antibiotic stewardship. Starting empirical antibiotic treatment immediately after surgical revision is recommended [26,27], since it has been shown to significantly improve treatment success when compared to delayed targeted antibiotic therapy following the surgical treatment of fracture-related infections [28]. However, the typically used broad-spectrum empirical antibiotic treatment should be adapted to targeted treatment as early as possible [26] to avoid the adverse drug effects of commonly used antibiotics [15,29] and development of antibiotic resistance. For empirical antibiotic treatment started immediately after surgery, the international FRI Consensus Group recommends using broad-spectrum antibiotics, including a lipopeptide or a glycopeptide and an agent covering Gram-negative bacteria [26,27], which have been shown to cover over 95% of causative pathogens [30]. Under the assumption of a maximum TTP of 24 h in Gram-negative rods, as observed in our study, it could be considered whether antibiotic treatment could be de-escalated to cover a Gram-positive germ spectrum after 48 h if Gram-negative pathogens are not identified up to this point using the diagnostic algorithm presented in this study. By reducing the time of administration of broad-spectrum antibiotics, adverse effects and antibiotic resistance could be prevented. The idea of the early de-escalation of antibiotic therapy after the exclusion of certain bacteria on the basis of their TTP, even without the identification of the causative pathogen in bacterial cultures, is supported by growing evidence. Lambregts et al. have already suggested an early re-evaluation of empirical broad-spectrum antibiotic treatment based on the reported times to positivity of common pathogens in suspected bacteriemia cases [31]. Moreover, such a procedure was already proposed in a study on individuals with onco-hematological diseases and febrile neutropenia by Puerta-Alcalde et al. [32], in which all multi-resistant pathogens were also detected in the blood culture within 24 h. However, further studies would be needed to investigate the safety and efficacy of early de-escalation of empirical antibiotic treatment in FRI.

In addition to evaluating the efficiency of a diagnostic test for the rapid detection of bacteria, the TTP has shown its value as a parameter of virulence and bacterial load [33]. Evidence of this correlation in the field of musculoskeletal infections is not yet available. However, a potential correlation of TTP with pathogen virulence can be derived from the fact that pathogens with consistently short TTP, like S. aureus and Gram-negative bacteria, usually lead to an acute FRI, while microorganisms with longer TTP, like coagulase-negative Staphylococci or *Cutibacterium acnes*, were more frequently cultured from delayed or chronic infections in previous studies [34]. Moreover, the TTP has proven additional prognostic value as a predictor of treatment outcome, especially in bacteriemia [35] and endocarditis [18]. In a meta-analysis by Hsieh et al. [17], a short TTP in blood culture increased the risk for septic shock and mortality as an independent risk factor. However, the correlation of TTP with disease severity and treatment success in musculoskeletal infections has not been investigated much yet. Nonetheless, significant variability in the TTP was observed among different bacterial species both in this study and other published studies in the field of musculoskeletal infections [13,24], with *Staphylococcus aureus*, Gram-negative bacteria, and *Enterococcus* species ranging among the pathogens with the shortest TTP. At the same time, MRSA [36,37,38], Gram-negative rods [39], and *Enterococci* [40] have been identified as potential risk factors for treatment failure in PJI, indicating a potential, though not yet investigated correlation.

The advantage of using sonication to constantly obtain TTP values resides in the process itself. Sonication enables the liberation of sessile bacteria from biofilms on orthopedic implants, improving the detection through standard microbiological analysis of liquid cultures. Since precise determination of the TTP in clinical routine practice is only feasible by automatic detection of bacterial growth in blood culture bottles, only this method ensures the generation of reliable and reproducible TTP values. In contrast to inoculating the standard culture medium with tissue samples or swabs, where plates are checked for growth once daily in clinical practice and the TTP can only be specified in days after inoculation [13], the presented method provides more reliable and comparable information regarding exact times to the hour.

The results of this investigation are subject to several limitations, primarily those inherent to the retrospective nature of the study. Second, the time to positivity only relates to the time from the incubation of the blood culture bottles to the first evidence of microbial growth. The pre-analytic period is not assessed in this parameter and was, therefore, not included in this study. Third, this study did not investigate the impact of the TTP in the cultures on the treatment outcome in the included cases. However, particularly Gram-negative germs and S. aureus have proven to have a very short TTP and these exact germs were associated with poor outcomes in related research on periprosthetic joint infections [39]. Additionally, at our institution, patients routinely receive perioperative antibiotics prior to skin incision, even in FRI cases. Cefazolin is given at a dose of 2 g routinely and clindamycin is administered in patients with contraindications to cefazolin. It is important to note that the use of prophylactic perioperative antibiotics has been shown not to impact the sensitivity of intraoperative cultures in revision surgeries for periprosthetic joint infections [41,42,43]. However, the influence of preoperative antibiotic prophylaxis on the time to positivity of cultures is not clear, since there might be a reduction in bacterial loads which could influence the time to positivity of cultures [33].

## 5. Conclusions

Overall, this study is the first to report the time to positivity of microorganisms in sonication fluid-infused blood culture bottles. We found that using this diagnostic technique leads to positive cultures within 24 h in over 85% of cases. These findings underscore the importance of sonication in diagnosing implant-related infections and highlight the diversity of pathogens involved, which is crucial for targeted antimicrobial therapy and infection control strategies. Notably, no Gram-negative bacteria were detected with a latency of over 24 h. Considering this, it should be discussed if empirical antibiotic therapy could be de-escalated early based on excluding Gram-negative bacteria if they are not detected up to a certain time point and limiting antibiotic coverage to the Gram-positive spectrum even without evidence of the exact causative germ. Further research is needed to investigate the value of the TTP as a predictor of treatment outcome and the impact of early diagnosis and early de-escalation of antibiotic therapy on the treatment success of FRIs.

## Figures and Tables

**Table 1:** Patient demographic characteristics (*n* = 126). The values are given as mean and standard deviation for metric data and as the number of patients and percentage in parentheses for categorical data.

Age (years)	57.0 ± 17.4
Sex	
Male	86 (68.3%)
Female	40 (31.7%)
Comorbidities	
Diabetes mellitus	18 (11.6%)
PAOD	14 (9.0%)
Affected bone	
Tibia	51 (40.5%)
Foot	28 (22.2%)
Femur	15 (11.9%)
Spine	9 (7.1%)
Fibula	7 (5.6%)
Humerus	7 (5.6%)
Forearm	5 (4.0%)
Pelvis/Hip	5 (4.0%)

**Table 2:** Distribution of pathogens detected in sonication fluids (*n* = 122). MSSA = methicillin-susceptible *S. aureus*; MRSA = methicillin-resistant *S. aureus*.

Detected Pathogens		Number of Patients (%)
Gram-positive bacteria		
*S. aureus*		
	MSSA	49 (40.2%)
	MRSA	3 (2.5%)
*S. epidermidis*		27 (22.1%)
Other *Staphylococcus* spp.		17 (13.9%)
	*S. hominis*	7 (5.7%)
	*S. warneri*	2 (1.6%)
	*S. haemolyticus*	2 (1.6%)
	*S. pettenkoferi*	1 (0.8%)
	*S. caprae*	1 (0.8%)
	*S. pseudointermedius*	1 (0.8%)
	*S. capitis*	1 (0.8%)
	*S. arlettae*	1 (0.8%)
	*S. lugdunensis*	1 (0.8%)
*Streptococcus* spp.		
	*S. dysgalactiae*	9 (7.4%)
	*S. agalactiae*	2 (1.6%)
	*S. pyogenes*	1 (0.8%)
	Alpha-haemolytic	1 (0.8%)
*Enterococcus* spp.		26 (21.3%)
*Cutibacterium* spp.		2 (1.6%)
Others		6 (4.9%)
Gram-negative bacteria		
*Pseudomonas aeruginosa*		6 (4.9%)
*Proteus* spp.		5 (4.1%)
*E. coli*		4 (3.3%)
*Enterobacter* spp.		4 (3.3%)
*Klebsiella* spp.		3 (2.5%)
*Morganella* spp.		3 (2.5%)
*Serratia* spp.		3 (2.5%)
*Sphingomonas* spp.		1 (0.8%)

**Table 3:** Time to positivity depending on the type of implant that was removed during revision surgery and sent for sonication.

	Spinal Rods (*n* = 6)	IMN (*n* = 15)	Screws (*n* = 94)	Plates (*n* = 44)	PMMA (*n* = 4)
PED	8.0 (IQR 7.4–9.3)	12.2 (IQR 7.4–18.5)	12.5 (IQR 9.3–17.1)	13.1 (IQR 9.8–20.2)	21.7 (IQR 7.1–24.7)
anaerobic	11.2 (IQR 8.3–21.6)	10.9 (IQR 7.3–16.1)	12.1 (IQR 10.1–19.1)	14.2 (IQR 10.5–19.6)	26.2 (IQR 22.1–112.4)

## Data Availability

The data presented in this study are available on request from the corresponding author. The data are not publicly available due to reasons of data protection.
